# Medical Causes of Hospitalisation among Patients with Bronchiectasis: A Nationwide Study in Japan

**DOI:** 10.3390/pathogens13060492

**Published:** 2024-06-09

**Authors:** Akihiko Hagiwara, Hisayuki Shuto, Ryohei Kudoh, Shota Omori, Kazufumi Hiramatsu, Jun-ichi Kadota, Kiyohide Fushimi, Kosaku Komiya

**Affiliations:** 1 Respiratory Medicine and Infectious Diseases, Oita University Faculty of Medicine, 1-1 Idaigaoka, Hasama-machi, Yufu 879-5593, Oita, Japan; 2 Department of Health Policy and Informatics, Tokyo Medical and Dental University Graduate School, 1-5-45 Yushima, Bunkyo-ku, Tokyo 113-8519, Japan; 3 Research Center for GLOBAL and LOCAL Infectious Diseases, Oita University Faculty of Medicine, 1-1 Idaigaoka, Hasama-machi, Yufu 879-5593, Oita, Japan

**Keywords:** bronchiectasis, post-infection, non-tuberculous mycobacterium, pneumonia

## Abstract

Purpose: Although the international guidelines for managing bronchiectasis are centred on preventing the exacerbation of bronchiectasis, the medical causes of admissions to hospital among patients with bronchiectasis have not been fully investigated. Methods: This study targeted patients with bronchiectasis who were admitted to hospitals between April 2018 and March 2020 using the national inpatient database in Japan. The causes of hospitalisation and types of antibiotics used for hospitalised patients were recorded. Results: In total, 21,300 hospitalisations of 16,723 patients with bronchiectasis were analysed. The most common cause was respiratory diseases in 15,145 (71.1%) admissions, including bacterial pneumonia and the exacerbation of bronchiectasis in 6238 (41.2%) and 3151 (20.8%), respectively. Antipseudomonal antibiotics were used in approximately 60% of patients with bacterial pneumonia who were administered antibiotic treatments and in approximately 50% of patients with the exacerbation of bronchiectasis. Conclusions: Bacterial pneumonia was the most frequent cause of hospitalisation, followed by the exacerbation of bronchiectasis, among patients with bronchiectasis. Physicians need to focus on the prevention of bacterial pneumonia in addition to the exacerbation of bronchiectasis in patients with bronchiectasis.

## 1. Introduction

Bronchiectasis is a chronic lung disease that is characterised by productive cough and recurrent infectious exacerbations with radiologically permanent and abnormal airway dilation [1,2]. Systemic symptoms, including fatigue, fever and weight loss, can be observed in advanced cases [3]. Recurrent exacerbations or physical deterioration with these symptoms may lead to a worsened prognosis with a decreased quality of life and increased healthcare costs [4,5].

Various underlying diseases may cause bronchiectasis. Reportedly, in a systematic review of 8608 patients with bronchiectasis, idiopathic bronchiectasis accounted for 44.8% of the patients, followed by post-infection (29.9%), immunodeficiency (5.0%), chronic obstructive pulmonary disease (COPD) (3.9%), connective tissue disease (3.8%), ciliary dysfunction (2.5%) and allergic bronchopulmonary aspergillosis (2.6%) [6]. However, the aetiology of bronchiectasis substantially varies with geographic region [7,8]. In Japan, a single institutional analysis revealed that idiopathic bronchiectasis accounted for 34% of patients with bronchiectasis, followed by sinobronchial syndrome (25%), non-tuberculous mycobacteriosis (18%) and a history of respiratory infection (14%) [9]. A recent registry study conducted in Japan revealed that non-tuberculosis mycobacteriosis accounted for approximately 80% of aetiology in bronchiectasis cases [10]. The prevalence of bronchiectasis is increasing worldwide and is reportedly higher among women and older people. For example, the prevalence of bronchiectasis in the United Kingdom increased from 350.5 cases to 566.1 cases and from 301.2 cases to 485.5 per 100,000 between 2004 and 2013 in women and men [11]. In addition, other studies from different countries revealed similar results [12,13,14,15,16].

The British Thoracic Society (BTS) and European Respiratory Society (ERS) guidelines recommend long-term oral macrolides or inhaled antibiotics in patients with three or more exacerbations per year in addition to airway clearance [5,17]. Recently, the airway microbiome, including the resistome characterised by diverse antimicrobial resistance genes, has been reported to be associated with clinical outcomes among patients with bronchiectasis [18,19]. This microbiome can be modified by long-term oral macrolides or inhaled antibiotics. Although these guidelines focus on preventing the exacerbations of bronchiectasis, the medical causes of hospitalisations other than exacerbations remain unclear. To determine what physicians should target for managing bronchiectasis, the causes of hospitalisation among patients with bronchiectasis should be determined. By collecting clinical data on hospitalisation among inpatients with bronchiectasis, potential interventions to reduce the future risk of hospitalisation and mortality should be considered.

While studies conducted in Europe and the United States have focused on the epidemiology of bronchiectasis [6,7,8,11,12,20], the medical causes of hospitalisation among patients with bronchiectasis have not been completely investigated. A few studies in Germany and the USA assessed the characteristics of hospitalisation in patients with bronchiectasis, but these studies focused on complications or secondary diagnoses among patients who were hospitalised due to the exacerbation of bronchiectasis [14,21]. As patients with bronchiectasis may be hospitalised for various reasons other than the exacerbation of bronchiectasis, large-scale studies are required to clarify the causes of hospitalisation, mortality rates and associated medical costs. This study used national datasets from the Japanese Diagnosis Procedure Combination (DPC) database, which provides data on most hospitalisations for acute illnesses in Japan [22]. The purpose of this study was to determine the medical causes of hospitalisation among patients with bronchiectasis in Japan.

## 2. Methods

### 2.1. Data Source

This study used national datasets obtained from the DPC database, which is a national inpatient database in Japan. The DPC system has been applied to 1764 hospitals (approximately 480,000 beds) as of 2022 and covers most hospitalisations for acute illnesses in Japan [22,23]. This database includes patient data on sex, age, body height and weight, diagnoses (main diagnosis, admission-precipitating diagnosis, most resource-consuming diagnosis, second most resource-consuming diagnosis, comorbidities present at admission and conditions arising post-admission) recoded with the International Classification of Diseases, 10th Revision (ICD-10) codes, procedures, drug administration, state of consciousness (Japan Coma Scale) at admission, Barthel Index, Charlson Comorbidity Index, smoking history (pack-years), length of hospitalisation stay, total hospitalisation costs and discharge status.

This study was approved by the Institutional Ethics Committee of Oita University, Faculty of Medicine (approval no. 2765, 22 March 2024). All aspects of the study comply with the Helsinki Declaration. The need for informed consent was waived by the committee considering the retrospective design of the study. Information on this research was posted at the hospital.

### 2.2. Study Population

We targeted patients with bronchiectasis admitted to DPC hospitals between April 2018 and March 2020. Patients who had an ICD-10 code of A162 or J47, which indicates bronchiectasis in their main diagnosis, admission-precipitating diagnosis, most resource-consuming diagnosis, second most resource-consuming diagnosis or comorbidities present at admission, were included in the analysis. Patients younger than 18 years of age were excluded.

### 2.3. Data Collection

The information of the patients, including sex, age, body mass index (BMI), smoking history (pack-years), Barthel Index, Charlson Comorbidity Index, state of consciousness, length of hospital stay, total hospitalisation costs (USD 1 equivalent to JPY 150) and in-hospital mortality, was collected from the DPC database. BMI was classified into four categories: <18.5, 18.5–24.9, 25.0–29.9 and ≥30.0 kg/m^2^; the Barthel Index was classified into five categories: <20, 20–39, 40–59, 60–79 and 80–100; and the Charlson Comorbidity Index was classified into four categories: 0, 1–2, 3–4 and ≥5. These classifications were in accordance with the international criteria and previous publications [24,25,26,27]. The state of consciousness was evaluated using the Japan Coma Scale, which is widely used to assess a patient’s consciousness level in Japan and considerably correlates with the Glasgow Coma Scale [28]. Impaired consciousness was defined as status other than zero assessed by the Japan Coma Scale in this study.

We determined the causes of hospitalisation among patients with bronchiectasis, referring to the ‘admission-precipitating diagnosis’ recoded in the ICD-10 code and Japanese disease names. They were categorised into major diagnostic categories, and patients admitted for the treatment of respiratory diseases were further classified. This study distinguished ‘pneumonia’ from ‘exacerbation of bronchiectasis’, which were recorded as an ‘admission-precipitating diagnosis’ in the DPC database. However, some patients with ‘exacerbation of bronchiectasis’ may also have ‘pneumonia’ as a complication. While the causes of hospitalisation were simply extracted from the ‘admission-precipitating diagnosis’, the proportion of ‘pneumonia’ complications that were recorded in diagnoses other than the ‘admission-precipitating diagnosis’ among patients admitted due to ‘exacerbation of bronchiectasis’ was also documented. Furthermore, viral infections, asthma and COPD, which were recorded as an ‘admission-precipitating diagnosis’, were not included under ‘exacerbation of bronchiectasis’ because the attending physicians appeared determined that ‘exacerbation of bronchiectasis’ was not the main cause of hospitalisation for these patients.

Data regarding the use of antibiotics, antifungals and inhaled drugs in the hospitalised patients were collected. We investigated the use of antibiotics listed in the Japanese Respiratory Society Guidelines for the Management of Pneumonia in Adults 2017 [29], particularly penicillins, such as amoxicillin (AMPC), ampicillin (ABPC), clavulanic acid/amoxicillin (CVA/AMPC), sultamicillin (SBTPC), benzylpenicillin (PCG), piperacillin (PIPC), sulbactam/ampicillin (SBT/ABPC) and tazobactam/piperacillin (TAZ/PIPC); cephalosporins, such as cefazolin (CEZ), cefotiam (CTM), cefcapene pivoxil (CDTR-PI), ceftriaxone (CTRX), cefotaxime (CTX), ceftazidime (CAZ), cefepime (CFPM), cefozopran (CZOP) and cefpirome (CPR); macrolides, such as clarithromycin (CAM), azithromycin (AZM) and erythromycin (EM); carbapenems, such as imipenem/cilastatin (IPM/CS), panipenem/betamipron (PAPM/BP), meropenem (MEPM), biapenem (BIPM) and doripenem (DRPM); tetracyclines, such as minocycline (MINO); quinolones, such as tosufloxacin (TFLX), levofloxacin (LVFX), garenoxacin mesilate hydrate (GRNX), moxifloxacin (MFLX), sitafloxacin (STFX), ciprofloxacin (CPFX) and pazufloxacin mesilate (PZFX); lincomycins, such as clindamycin (CLDM); nitroimidazoles, such as metronidazole (MNZ); glycopeptides, such as vancomycin (VCM) and teicoplanin (TEIC); oxazolidinones, such as linezolid (LZD); and aminoglycosides, such as gentamicin (GM), amikacin (AMK), tobramycin (TOB) and arbekacin (ABK). The following antibiotics were defined as antipseudomonal antibiotics—which are typically considered susceptible to *Pseudomonas aeruginosa* (*P. aeruginosa)*: PIPC, TAZ/PIPC, CAZ, CFPM, CZOP, CPR, IPM/CS, PAPM/BP, MEPM, BIPM, DRPM, TFLX, LVFX, STFX, CPFX, PZFX, GM, AMK and TOB. For antifungals, the use of amphotericin B (AMPH-B), liposomal amphotericin B (L-AMB), micafungin (MCFG), caspofungin acetate (CPFG), fluconazole (FLCZ), fosfluconazole (F-FLCZ), itraconazole (ITCZ) and voriconazole (VRCZ) was evaluated. As the DPC database contains information limited to medications prescribed during hospitalisation, the status of chronic or acute medication before admission could not be obtained.

### 2.4. Statistical Analysis

Statistical analyses were performed using IBM SPSS Statistics for Windows version 28 (IBM Japan, Tokyo, Japan). The values are presented as median with interquartile range for continuous variables and number with proportion for categorical variables in the analysis of the overall patient characteristics, distribution of admission causes among patients with bronchiectasis, distribution of respiratory disease-related hospitalisations and use of antibiotics and antifungals for bacterial pneumonia and the exacerbation of bronchiectasis.

## 3. Results

### 3.1. Patients’ Characteristics

Between April 2018 and March 2020, 21,300 hospitalisations of 16,723 patients with bronchiectasis were identified. Table 1 shows their characteristics at admission. Of the 21,300 hospitalisations, the median age was 77 years (interquartile range [IQR], 70–83 years), 14,122 (66.3%) were female, and 9115 (42.8%) had BMI < 18.5. The median length of hospital stay, total hospitalisation costs and in-hospital mortality were 13 days (IQR: 8–22), USD 3889 (IQR: 2366–6883) and 7.2% (1528/21,300), respectively.

### 3.2. Cause of Hospitalisation

The most common reason for admission was respiratory diseases, associated with 15,145 (71.1%) hospitalisations, followed by malignancies in 1043 (4.9%) and cardiovascular diseases in 998 (4.7%) (Table 2). Cardiovascular diseases had the highest in-hospital mortality rate, and heart failure accounted for 69.1% (65/94) of cardiovascular disease-related mortality. ‘Others’ also had a high in-hospital mortality rate; however, this category included 65 cases of cardiopulmonary arrest, and 56 patients died during hospitalisation. Musculoskeletal diseases had the longest hospitalisation length, and bone fracture accounted for 67.7% (627/926) of the category. Of the 15,145 respiratory disease-related hospitalisations, the major causes of hospitalisation were bacterial pneumonia in 6238 (41.2%), the exacerbation of bronchiectasis in 3151 (20.8%) and haemoptysis in 1595 (10.5%) (Table 3). Complications of pneumonia, recorded as ‘pneumonia’ in diagnoses other than the ‘admission-precipitating diagnosis’, were observed in 24.8% (782/3151) of the patients with the exacerbation of bronchiectasis and in 19.3% (308/1595) of the patients with haemoptysis. Of 657 (4.3%) admissions due to respiratory failure, bacterial pneumonia and the exacerbation of bronchiectasis were associated with 123 and 151 admissions, respectively. ‘Upper airway infection’ recoded as the ‘admission-precipitating diagnosis’ was found in only seven cases. Although 132 patients were hospitalised because of influenza infection, no patients were admitted for respiratory syncytial virus or coronavirus infections. Lung cancer ranked fifth among the causes of respiratory disease-related hospitalisations. The most resource-consuming diagnosis among these patients was also ‘lung cancer’, observed in 94.5% (742/785), indicating that most hospitalisations were to diagnose or treat lung cancer rather than non-lung cancer–associated diseases, such as infectious diseases.

### 3.3. The Use of Antibiotics, Antifungals and Inhaled Drugs

Antibiotics were used in 6132 (98.3%) of 6238 bacterial pneumonia-related admissions and in 2496 (79.2%) of 3151 cases of exacerbation of bronchiectasis-related admissions (Table 4). Moreover, antifungals were used in a few patients. The most frequently used antibiotics for bacterial pneumonia were penicillins (63.1%), followed by cephalosporins (42.7%), macrolides (35.5%), quinolones (20.8%) and carbapenems (14.2%). Macrolides (65.2%), followed by penicillins (42.9%), cephalosporins (34.4%), quinolones (19.1%) and carbapenems (10.6%), were frequently used for the exacerbation of bronchiectasis. Antipseudomonal antibiotics were administered to 62.6% (3840/6132) of the patients hospitalised because of bacterial pneumonia who underwent antibiotic treatment, as well as 49.4% (1233/2496) of the patients hospitalised because of the exacerbation of bronchiectasis. Any inhaled drugs were used in 26.9% (1679/6238) of the cases of bacterial pneumonia-related admission: 91 (5.4%) with an inhaled corticosteroid; 85 (5.1%) with a long-acting β2 agonist; 266 (15.8%) with a long-acting muscarinic antagonist; 491 (29.2%) with an inhaled corticosteroid and a long-acting β2 agonist; 291 (17.3%) with a long-acting β2 agonist and a long-acting muscarinic antagonist; 17 (1.0%) with an inhaled corticosteroid, a long-acting β2 agonist and a long-acting muscarinic antagonist; 877 (52.2%) with a short-acting β2 agonist; and 4 (0.2%) with a short-acting muscarinic antagonist. In contrast, any inhaled drugs were used in 24.7% (777/3151) of the cases of exacerbation of bronchiectasis-related admission: 63 (8.1%) with an inhaled corticosteroid; 52 (6.7%) with a long-acting β2 agonist; 176 (22.7%) with a long-acting muscarinic antagonist; 245 (31.5%) with an inhaled corticosteroid and a long-acting β2 agonist; 154 (19.8%) with a long-acting β2 agonist and a long-acting muscarinic antagonist; 9 (1.2%) with an inhaled corticosteroid, a long-acting β2 agonist and a long-acting muscarinic antagonist; 312 (40.2%) with a short-acting β2 agonist; and 4 (0.5%) with a short-acting muscarinic antagonist. None of the patients used inhaled antibiotics in this study.

## 4. Discussion

This study shows that respiratory diseases accounted for approximately 70% of admission causes among patients with bronchiectasis, in which bacterial pneumonia was clearly the most common reason. Given that respiratory diseases account for 6% of hospitalisations among the general population in Japan [30], patients with bronchiectasis are at high risk of admission due to respiratory diseases. The pathobiological mechanism of bronchiectasis involves a vicious cycle of chronic bronchial infection, inflammation, impaired mucociliary clearance and structural lung damage [5,31], which may affect susceptibility to bacterial pneumonia and the acute exacerbation of bronchiectasis. Indeed, this study found that approximately a quarter of patients were hospitalised due to ‘exacerbation of bronchiectasis’ complicated with ‘pneumonia’. It is unclear whether physicians clearly discriminated between ‘exacerbation of bronchiectasis’ and ‘pneumonia’ in this DPC database analysis. Nevertheless, pneumonia should be considered a major cause of hospitalisation among patients with bronchiectasis.

Long-term macrolide therapy is effective in preventing acute exacerbations of bronchiectasis; however, its efficacy in preventing pneumonia development remains unclear [32,33]. This database study could not technically assess the association between the use of long-term macrolides before hospitalisation and the risk of pneumonia development. As a typical strategy for pneumonia prevention, influenza and pneumococcal vaccines are recommended for elderly people or immunocompromised individuals [29,34]. Although the effectiveness of preventing pneumonia specifically in patients with bronchiectasis has not been investigated, several trials have shown that these vaccines reduced the risk of exacerbations of COPD and asthma [35,36,37,38,39]. Because these diseases are a part of the underlying diseases associated with bronchiectasis, the evidence of vaccine efficacy might apply to bronchiectasis.

Herein, antipseudomonal antibiotics were used in approximately 60% of the patients with bacterial pneumonia who underwent antibiotic treatment. *Pseudomonas aeruginosa* is frequently isolated from respiratory samples of patients with bronchiectasis. The European Multicentre Bronchiectasis Audit and Research Collaboration showed that 3047 (25.1%) of 12,152 patients with bronchiectasis had cultures positive for *P. aeruginosa* [8]. Reportedly, the presence of chronic infection with *P. aeruginosa* is associated with a poor prognosis [40,41]. However, it was not feasible to determine whether *P. aeruginosa* was isolated from the samples of patients included in our study because the DPC database does not contain microbiological data. It remains unclear whether the use of antipseudomonal antibiotics is appropriate for pneumonia that develops in patients with bronchiectasis.

The exacerbation of bronchiectasis was the second most common cause of hospitalisation. Long-term macrolide therapy is recommended for patients with three or more exacerbations per year according to the BTS and ERS guidelines [5,17]. Macrolides were frequently used for patients with bronchiectasis who were hospitalised. However, it could not be determined by the DPC database whether these patients were treated with macrolides pre-admission or newly received macrolides during hospitalisation. Some patients with bronchiectasis were hospitalised due to asthma or COPD. Interestingly, 65.5% (116/177) of the patients with asthma and 75.9% (126/166) of those with COPD were treated with macrolides during hospitalisation. There is evidence that long-term macrolide therapy is effective in preventing exacerbation in patients with advanced asthma and COPD, with or without bronchiectasis [42,43,44]. Although the severity of asthma and COPD could not be extracted from the DPC database, macrolide therapy might have been considered for both disease severity and bronchiectasis complications.

Antipseudomonal antibiotics were most commonly used for patients with the exacerbation of bronchiectasis who underwent antibiotic treatment in this study. The BTS guidelines suggest the use of antipseudomonal antibiotics for the exacerbation of bronchiectasis if *P. aeruginosa* is detected in previous tests [17]. Reportedly, exacerbations are involved in the respiratory dysbiosis of the microbial ecosystem of the respiratory tract, and the isolation of drug-resistant *P. aeruginosa* during exacerbations is not necessarily associated with the clinical course, which is different from that of acute bacterial infections, including pneumonia [45]. Similarly, with pneumonia, it has not been determined whether *P. aeruginosa* should be targeted in patients with an acute exacerbation of bronchiectasis when this pathogen is isolated from respiratory samples. Whether or not *P. aeruginosa* isolated from patients with bronchiectasis should be targeted is a research question to be addressed.

Malignancies, cardiovascular diseases, digestive diseases and musculoskeletal diseases were the major causes of hospitalisation other than respiratory diseases. Cardiovascular diseases had the highest in-hospital mortality rate (Table 2), and heart failure accounted for two-thirds of cardiovascular disease-related mortality. Considering that the hospital cost in the cardiovascular disease group was higher than that in the respiratory disease group despite a similar length of hospital stay, patients who were hospitalized due to cardiovascular diseases possibly suffered from severe conditions, including coronary artery diseases that required expensive interventions. Musculoskeletal diseases had the longest hospital stay duration. As bone fractures were the major diseases classified in this category, a longer hospital stay would be required because of surgical interventions and subsequent rehabilitations [46,47]. The proportion of these diseases as hospitalisation causes is possibly lower than that in the general population because respiratory diseases accounted for most hospitalisations. However, approximately 30% of deaths in patients with bronchiectasis were reportedly associated with events other than respiratory diseases [48], which implies the need for comorbidity management. As with the concept of COPD being regarded as a systemic disease, bronchiectasis should also be considered a systemic disease.

Lung cancer is a major cause of death among malignant diseases [49]. Nevertheless, the mortality in patients with malignancies, except for lung cancer, was lower than that in patients with lung cancer. The DPC database collects information for each hospitalisation and not for each individual patient. Patients with lung cancer are more frequently hospitalised for diagnosis or repeated therapies than those with other malignancies [50]. The in-hospital mortality rate of pulmonary tuberculosis was 7.6%, denoting 34.6 per 100,000 hospitalised patients with bronchiectasis. In accordance with nationwide statistics in 2019, the mortality of tuberculosis in Japan was approximately 1.7 per 100,000 people [51]. Although the mortality between these groups could not be compared because of different patient backgrounds, the mortality in patients with bronchiectasis might be higher than that in the general population.

The strength of this study is that it focused on the hospitalisation causes of patients with bronchiectasis using a nationwide database in Japan. While the guidelines focus on the prevention of the acute exacerbation of bronchiectasis, bacterial pneumonia was found to be the most frequent cause of hospitalisation among patients with bronchiectasis. However, this study had several limitations. First, the patients included in this study might not necessarily have met the international diagnostic criteria because the DPC database does not guarantee the reliability and validity of the diagnosis. For example, it was uncertain whether physicians accurately diagnosed pneumonia based on clinical symptoms and chest images, and it was not known how they distinguished ‘exacerbation of bronchiectasis’ from ‘pneumonia’. Similarly, the exacerbation of asthma and COPD might not be sufficiently distinguished from ‘exacerbation of bronchiectasis’. Although this study simply extracted the cause of hospitalisation from the name of ‘admission-precipitating diagnosis’, such limitations are commonly observed in large dataset analyses. Second, we enrolled only hospitalised patients with bronchiectasis based on the DPC database diagnosis according to the ICD-10 code, thus potentially underestimating the total number of patients with bronchiectasis. Patient background, such as disease severity, might vary between inpatients and outpatients, and different results could have been obtained if outpatients were included. Third, we could not directly assess the risk of hospitalisation due to bacterial pneumonia compared with the risk in the general population. The frequency of hospitalisation due to respiratory diseases appeared high among patients with bronchiectasis; however, this could not be statistically compared. Fourth, the results might be affected by patient background and medical care system, as this study used an inpatient database in Japan. For example, over more than half of the patients with bronchiectasis who were hospitalised in Germany were <65 years old [14], and the median age was 71 years in men and 75 years in women hospitalised in the USA [21]. Hence, the current results should be cautiously generalised to other countries.

## 5. Conclusions

Bacterial pneumonia was the most frequent cause of hospitalisation by a large margin, followed by the exacerbation of bronchiectasis, among patients with bronchiectasis. Physicians should focus on the prevention of bacterial pneumonia in addition to the exacerbation of bronchiectasis in these patients.

## Figures and Tables

**Table 1:** Patient characteristics at admission (N = 21,300).

Variables	Number (%) or Median (Interquartile Range)
Age (years)	77 (70–83)
Sex (female)	14,122 (66.3)
BMI (kg/m^2^)	
<18.5	9115 (42.8)
18.5–24.9	9316 (43.7)
25.0–29.9	1351 (6.3)
≥30.0	225 (1.1)
Missing data	1293 (6.1)
Smoking history	
Non-smoker	15,198 (71.4)
Current/past smoker	4225 (19.8)
Missing data	1877 (8.8)
Barthel index	
<20	3056 (14.3)
20–39	801 (3.8)
40–59	1635 (7.7)
60–79	1538 (7.2)
80–100	11,832 (55.5)
Missing data	2438 (11.4)
Charlson Comorbidity Index	
0	1914 (9.0)
1–2	13,928 (65.4)
3–4	4645 (21.8)
≥5	813 (3.8)
Impaired consciousness	2479 (11.6)
Hospital stay (days)	13.0 (8.0–22.0)
Hospitalisation cost (USD)	3889 (2366–6883)
In-hospital mortality	1528 (7.2)

Values are presented as median (interquartile range) or n (%); BMI, body mass index.

**Table 2:** Distribution of admission causes among patients with bronchiectasis (N = 21,300).

Cause of Hospitalisation	Number (%)	Age (Years)	Sex (Female)	Hospital Stay (Days)	Hospitalisation Cost (USD)	In-Hospital Mortality
Respiratory diseases	15,145 (71.1)	77 (69–83)	10,046 (66.3)	13 (8–22)	3718 (2363–6141)	1119 (7.4)
Malignancy	1043 (4.9)	75 (69–81)	565 (54.2)	11 (7–22)	4955 (2836–9494)	68 (6.5)
Cardiovascular diseases	998 (4.7)	79 (73–86)	641 (64.2)	13 (5–24)	5675 (2911–10,561)	94 (9.4)
Digestive diseases	932 (4.4)	77 (71–83)	552 (59.2)	9 (5–15)	3163 (1803–5145)	32 (3.4)
Musculoskeletal diseases	926 (4.3)	81 (76–87)	768 (82.9)	26 (15–49)	9257 (4875–13,720)	39 (4.2)
Brain diseases	384 (1.8)	80 (73–85)	248 (64.6)	16 (9–39)	6367 (2995–12,049)	31 (8.1)
Kidney and urinary tract diseases	332 (1.6)	79 (71–85)	224 (67.5)	14 (8–24)	3735 (2500–6198)	19 (5.7)
Connective tissue diseases	222 (1.0)	73 (67–78)	160 (72.1)	16 (3–32)	4392 (1186–10,149)	5 (2.3)
Ear, nose and throat diseases	138 (0.6)	75 (65–81)	97 (70.3)	8 (5–11)	2838 (1509–5966)	1 (0.7)
Eye diseases	127 (0.6)	77 (70–83)	93 (73.2)	3 (2–5)	1673 (1484–2945)	1 (0.8)
Blood diseases	92 (0.4)	78 (70–85)	64 (69.6)	11 (6–20)	3316 (1919–5800)	8 (8.7)
Skin diseases	83 (0.4)	77 (69–86)	60 (72.3)	13 (8–20)	3318 (1848–4807)	2 (2.4)
Endocrine, nutritional and metabolic diseases	80 (0.4)	78 (68–82)	49 (61.3)	12 (8–21)	3407 (2082–4814)	1 (1.3)
Gynaecological diseases	39 (0.2)	59 (41–78)	39 (100.0)	7 (5–10)	4251 (1773–5949)	0 (0.0)
Others	759 (3.6)	79 (72–85)	516 (68.0)	12 (6–23)	3537 (1917–6426)	108 (14.2)

Values are presented as median (interquartile range) or n (%).

**Table 3:** Distribution of respiratory disease-related hospitalisation (N = 15,145).

Cause of Hospitalisation	Number (%)	Age (Years)	Sex (Female)	Hospital Stay (Days)	Hospitalisation Cost (USD)	In-Hospital Mortality
Bacterial pneumonia	6238 (41.2)	79 (71–85)	4185 (67.1)	15 (10–24)	3832 (2702–6068)	585 (9.4)
Exacerbation of bronchiectasis	3151 (20.8)	76 (68–82)	2233 (70.9)	12 (7–19)	3359 (2047–5524)	169 (5.4)
Haemoptysis	1595 (10.5)	76 (69–81)	1059 (66.4)	9 (6–14)	3615 (2264–5851)	38 (2.4)
Pulmonary non-tuberculous mycobacteria	924 (6.1)	73 (65–79)	773 (83.7)	9 (3–18)	2561 (1132–4711)	31 (3.4)
Lung cancer	785 (5.1)	72 (68–78)	350 (44.6)	6 (3–15)	4776 (2785–9390)	23 (2.9)
Respiratory failure	657 (4.3)	76 (70–82)	438 (66.7)	18 (10–34)	5323 (3326–9511)	134 (20.4)
Interstitial lung disease	416 (2.7)	76 (70–81)	251 (60.3)	15 (8–25)	4237 (2485–6569)	36 (8.7)
Pneumothorax	211 (1.4)	73 (66–79)	110 (52.1)	15 (10–26)	4645 (2652–8275)	12 (5.7)
Pulmonary mycosis	190 (1.3)	71 (62–78)	101 (53.2)	15 (8–23)	4624 (2653–7922)	16 (8.4)
Asthma	177 (1.2)	75 (63–81)	137 (77.4)	10 (6–18)	3172 (2168–4538)	1 (0.6)
COPD	166 (1.1)	77 (71–81)	59 (35.5)	14 (9–25)	4214 (2695–6778)	21 (12.7)
Lung abscess	109 (0.7)	71 (64–79)	55 (50.5)	19 (12–32)	5504 (3761–9811)	7 (6.4)
Pulmonary tuberculosis	105 (0.7)	79 (68–86)	62 (59.0)	12 (5–28)	3322 (1633–6550)	8 (7.6)
Empyema	74 (0.5)	75 (71–81)	20 (27.0)	22 (16–36)	6845 (4852–9325)	8 (10.8)
Diffuse panbronchiolitis	66 (0.4)	72 (62–80)	38 (57.6)	15 (10–23)	4318 (3042–6669)	4 (6.1)
Other respiratory disease	281 (1.9)	75 (65–81)	175 (62.3)	13 (7–22)	3937 (2237–7457)	26 (9.3)

Values are presented as median (interquartile range) or n (%); COPD, chronic obstructive pulmonary disease.

**Table 4:** Use of antibiotics and antifungals for bacterial pneumonia and the exacerbation of bronchiectasis.

Cause of Hospitalisation	Type of Antimicrobials	Number (%)	Name of Antibiotics (Number with %)
Bacterial pneumonia		6238 (100)	
	Use of antibiotics	6132 (98.3)	
	Penicillins	3871 (63.1)	TAZ/PIPC 2077 (53.6), SBT/ABPC 1708 (44.1), CVA/AMPC 442 (11.4)
	Cephalosporins	2620 (42.7)	CTRX 1730 (66.0), CAZ 469 (17.9), CFPM 310 (11.8)
	Macrolides	2178 (35.5)	CAM 1563 (71.8), EM 1456 (66.9), AZM 393 (18.0)
	Quinolones	1274 (20.8)	LVFX 809 (63.5), GRNX 252 (19.8), STFX 108 (8.5)
	Carbapenems	873 (14.2)	MEPM 754 (86.4), DRPM 120 (13.7), BIPM 13 (1.5)
	Aminoglycosides	172 (2.8)	AMK 106 (61.6), TOB 47 (27.2), GM 24 (13.9)
	Glycopeptides	154 (2.5)	VCM 141 (91.6), TEIC 20 (13.0)
	Tetracyclines	121 (2.0)	MINO 121 (100.0)
	Lincomycins	107 (1.7)	CLDM 107 (100.0)
	Nitroimidazoles	68 (1.1)	MNZ 68 (100.0)
	Oxazolidinones	22 (0.4)	LZD 22 (100.0)
	Use of antifungals	238 (3.8)	ITCZ 85 (35.7), VRCZ 66 (27.7), MCFG 63 (26.5)
Exacerbation of bronchiectasis		3151	
	Use of antibiotics	2496 (79.2)	
	Macrolides	1628 (65.2)	CAM 834 (51.2), EM 763 (46.9), AZM 100 (6.1)
	Penicillins	1071 (42.9)	TAZ/PIPC 520 (48.6), SBT/ABPC 445 (41.5), CVA/AMPC 129 (12.0)
	Cephalosporins	858 (34.4)	CTRX 411 (48.0), CAZ 195 (22.7), CFPM 142 (16.6)
	Quinolones	476 (19.1)	LVFX 238 (50.0), GRNX 103 (21.6), STFX 69 (14.5)
	Carbapenems	265 (10.6)	MEPM 224 (84.5), DRPM 31 (11.7), BIPM 10 (3.8)
	Aminoglycosides	97 (3.9)	AMK 61 (62.9), TOB 26 (26.8), GM 10 (10.3)
	Tetracyclines	26 (1.0)	MINO 26 (100.0)
	Glycopeptides	22 (0.9)	VCM 21 (95.5), TEIC 1 (0.5)
	Lincomycins	31 (1.2)	CLDM 31 (100.0)
	Nitroimidazoles	14 (0.6)	MNZ 14 (100.0)
	Oxazolidinones	3 (0.1)	LZD 3 (100.0)
	Use of antifungals	105 (3.3)	ITCZ 36 (34.3), VRCZ 34 (32.4), MCFG 25 (23.8)

Values are presented as n (%). AMK, amikacin; AZM, azithromycin; BIPM, biapenem; CAM, clarithromycin; CAZ, ceftazidime; CFPM, cefepime; CLDM, clindamycin; CTRX, ceftriaxone; CVA/AMPC, clavulanic acid/amoxicillin; DRPM, doripenem; EM, erythromycin; GM, gentamicin; GRNX, garenoxacin mesilate hydrate; ITCZ, itraconazole; LVFX, levofloxacin; LZD, linezolid; MCFG, micafungin; MEPM, meropenem; MINO, minocycline; MNZ, metronidazole; SBT/ABPC, sulbactam/ampicillin; STFX, sitafloxacin; TAZ/PIPC, tazobactam/piperacillin; TEIC, teicoplanin; TOB, tobramycin; VCM: vancomycin; VRCZ, voriconazole.

## Data Availability

The data are available from the corresponding author upon reasonable request.
